# A Partial Discharge Study of Medium-Voltage Motor Winding Insulation Under Two-Level Voltage Pulses With High Dv/Dt

**DOI:** 10.1109/ojpel.2021.3069780

**Published:** 2021-03-30

**Authors:** BOXUE HU, ZHUO WEI, HAOYANG YOU, RISHA NA, RUI LIU, HAN XIONG, PENGYU FU, JULIA ZHANG, JIN WANG

**Affiliations:** Department of Electrical and Computer Engineering, The Ohio State University, Columbus, OH 43210 USA

**Keywords:** Silicon carbide (SiC), pulse width modulation (PWM), electric machines, variable speed drives, partial discharge, high-voltage techniques

## Abstract

Medium-voltage (e.g., 10 kV rated) silicon carbide (SiC) devices have great potentials in medium-voltage variable speed drives. But their high switching dv/dt can increase the voltage stress on motor windings and cause partial discharges. This paper presents a partial discharge study of a medium-voltage form-wound winding under two-level square-wave voltage pulses. A 10 kV SiC device-based test platform is built to generate voltage pulses with high dv/dt. A three-step test approach is proposed and employed to systematically investigate the effects of various voltage parameters on partial discharges. These include voltage rise/fall time, voltage pulse width, pulse repetitive rate, duty ratio, voltage polarity, fundamental frequency, and modulation index. Partial discharge inception voltages (PDIVs) and repetitive partial discharge inception voltages (RPDIVs) of the sample are measured with varied voltage parameters. Test results show that voltage rise/fall time is a major affecting factor which reduces PDIVs of the winding sample by 6.5% when it decreases from 800 ns to 100 ns. Based on test results, a hypothetical partial discharge mechanism is presented to explain the effects of fast voltage rise/fall edges. An empirical equation is also derived to estimate PDIVs of a winding sample under various voltage rise/fall time and pulse width conditions.

## I. INTRODUCTION

Multilevel inverters based on silicon (Si) insulated-gate bipolar transistors (IGBTs) or thyristors have been widely researched and built as variable speed drives (VSDs) for medium-voltage electric machines to improve their efficiency and performance [1], [2]. Si devices with medium voltage ratings are limited in voltage ratings (generally less than 6.5 kV) and have excessive power loss when operated at switching frequencies over 1 kHz [3]. Emerging medium-voltage silicon carbide (SiC) devices outperform existing Si devices in voltage ratings, specific on-state resistances, switching speeds, and maximum allowable junction temperatures [4], [5]. These SiC devices enable potential implementations of simple three-phase two-level VSDs for medium-voltage electric machines [6], [7]. Applying SiC devices in medium-voltage VSDs can gain benefits including simplified circuit topologies and controls [6], [7], reduced power loss [6], [7], higher power density [8], [9], better power quality [6], [9], and faster dynamic response [6], [8].

High voltage ratings and fast switching speeds of SiC devices can increase the voltage stress on the insulation of motor windings. First, the reflected wave phenomenon in a SiC device-based motor drive is more severe than that in a Si device-based system due to the fact that SiC devices can switch higher voltages at faster speeds [10], [11]. Second, fast-switched voltages create uneven voltage distributions in motor winding insulations [12], [13]. For instance, it was reported that the peak voltage across the first winding turn could be more than five times of that on the last winding turn when a motor was driven by a SiC device-based VSD [12]. The increased voltage stress can induce partial discharges in motor windings.

Partial discharge is an electrical breakdown that bridges only a small portion of the insulation between two electrodes under high voltage stress [14]. It can degrade winding insulations and reduce motor lifetimes [15], [16]. To address partial discharges caused by the installations of VSDs, organizations such as International Electrotechnical Commission (IEC) and National Electrical Manufacturers Association (NEMA) have published standards to qualify motor insulations for their reliable operations with VSDs [17]-[19]. Researches were conducted on partial discharges in motor windings under voltage stress with various parameters, including voltage waveform shape [20]-[22], voltage polarity [23], voltage pulse repetitive rate [23], [24], and voltage rise/fall time [21], [24]-[27]. Most recent studies on partial discharges in motor windings associated with SiC device-based VSDs focus on low-voltage random-wound machines [25], [26]. Partial discharge studies on medium-voltage motor windings are mostly done for 50/60 Hz or Si device-based systems [21], [28]. For instance, reference [21] used 30 kV Si transistors to apply two-level and multilevel high-voltage pulses to motor winding samples. The 30 kV Si transistor has long switching rise/fall time (> 400 ns) and limited switching frequency (<1.5 kHz). While medium-voltage SiC devices are capable of switching within 100 ns with switching frequencies of 10 kHz [6], [7]. Moreover, existing studies often apply repetitive voltage pulses to the motor winding test sample to emulate the continuous voltage stress in real operations of VSDs. Test results obtained in this way are inevitably affected by interactions of voltage waveform parameters, accumulated charges [29], elevated temperatures of the test sample [27], etc.

Potential application of medium-voltage SiC devices in two-level VSDs will create unprecedented voltage stress (e.g., short rise time, high switching frequency, and two-level high voltage change) on medium-voltage motor windings which are not fully covered in previous partial discharge studies. It is necessary to identify the main influencing voltage parameters on partial discharges and to refine the existing test approach. This paper presents a partial discharge study of a form-wound motor winding, focusing on its phase-to-ground insulation under two-level voltage pulses. Contributions of this paper include:

1. A 10 kV SiC device-based partial discharge test platform is built to apply two-level voltage pulses with high dv/dt to the motor winding sample.
2. A three-step test approach is proposed and adopted to systematically investigate effects of different voltage waveform parameters on partial discharge inception voltages (PDIVs). The test approach reduces the effects of interactions of voltage waveform parameters, accumulated charges, and temperature increases of the test sample on test results.
3. Voltage rise/fall time is identified as the main influencing voltage waveform parameter on PDIVs. Explanations of effects of fast voltage rise/fall edges on partial discharge processes are presented. An empirical equation is also derived to estimate PDIVs of a winding sample under various voltage rise/fall time and pulse width conditions.

The rest of the paper proceeds as follows. Section II presents the partial discharge test platform. Section III introduces the three-step partial discharge test approach. Measurement results of PDIVs and repetitive partial discharge inception voltages (RPDIVs) of the winding sample under various test conditions are shown in section IV. Based on the test results, section V provides a hypothetical explanation on effects of fast voltage rise/fall edges and an empirical equation to estimate PDIVs of a motor winding sample. Section VI concludes this paper.

## II. PARTIAL DISCHARGE TEST PLATFORM

**FIGURE 1.**
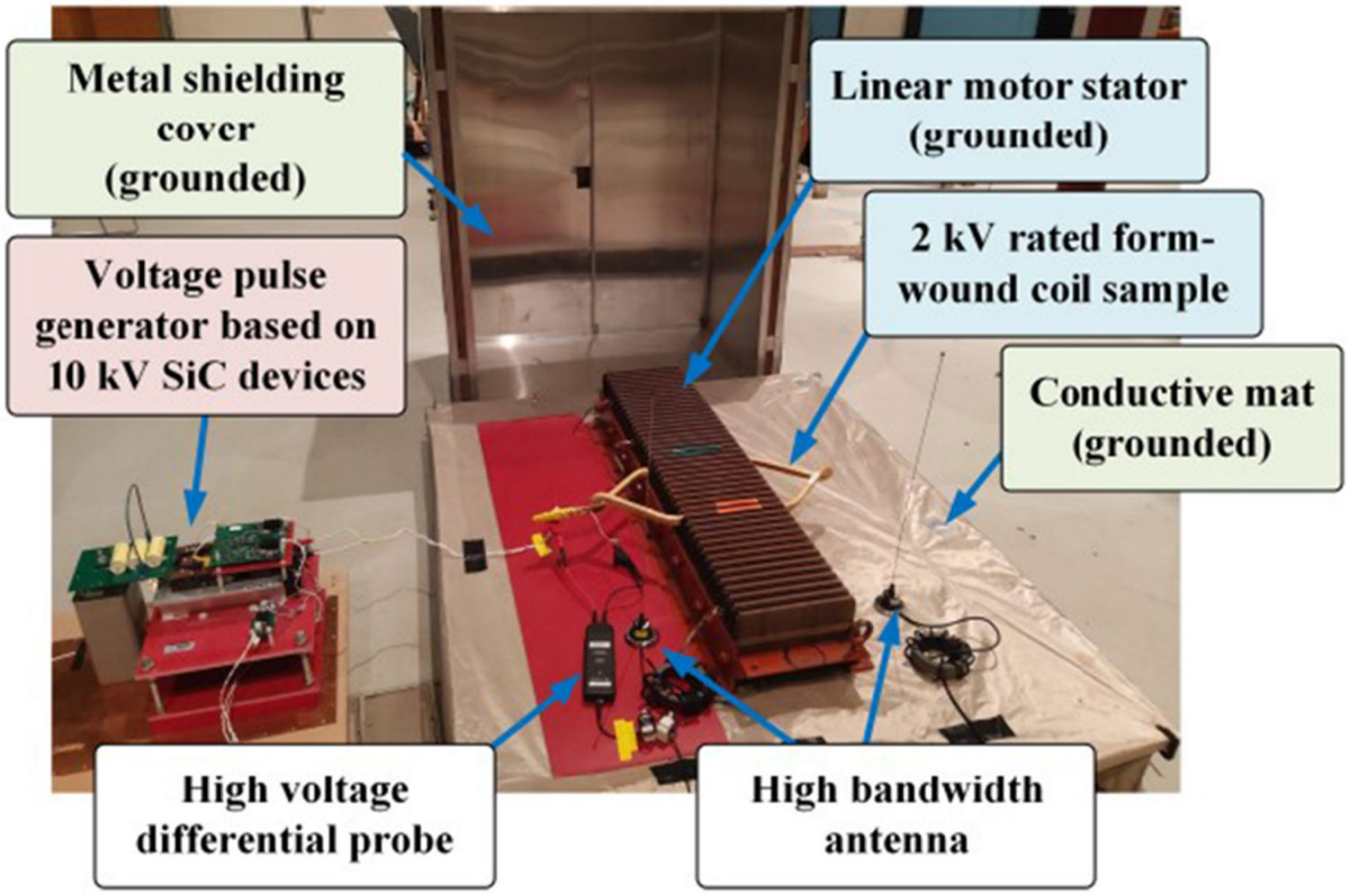
An overview of the partial discharge test platform.

An overview of the partial discharge test platform is shown in Fig. 1. It consists of four main parts: a voltage pulse generator, a motor winding sample, measurement devices, and a Faraday shield. The Faraday shield is formed by a metal shielding cover and a conductive mat. The winding sample and measurement devices are placed inside the grounded Faraday shield during tests to mitigate impacts of background noises and switching noises produced by the voltage pulse generator.

### A. VOLTAGE PULSE GENERATOR

**FIGURE 2.**
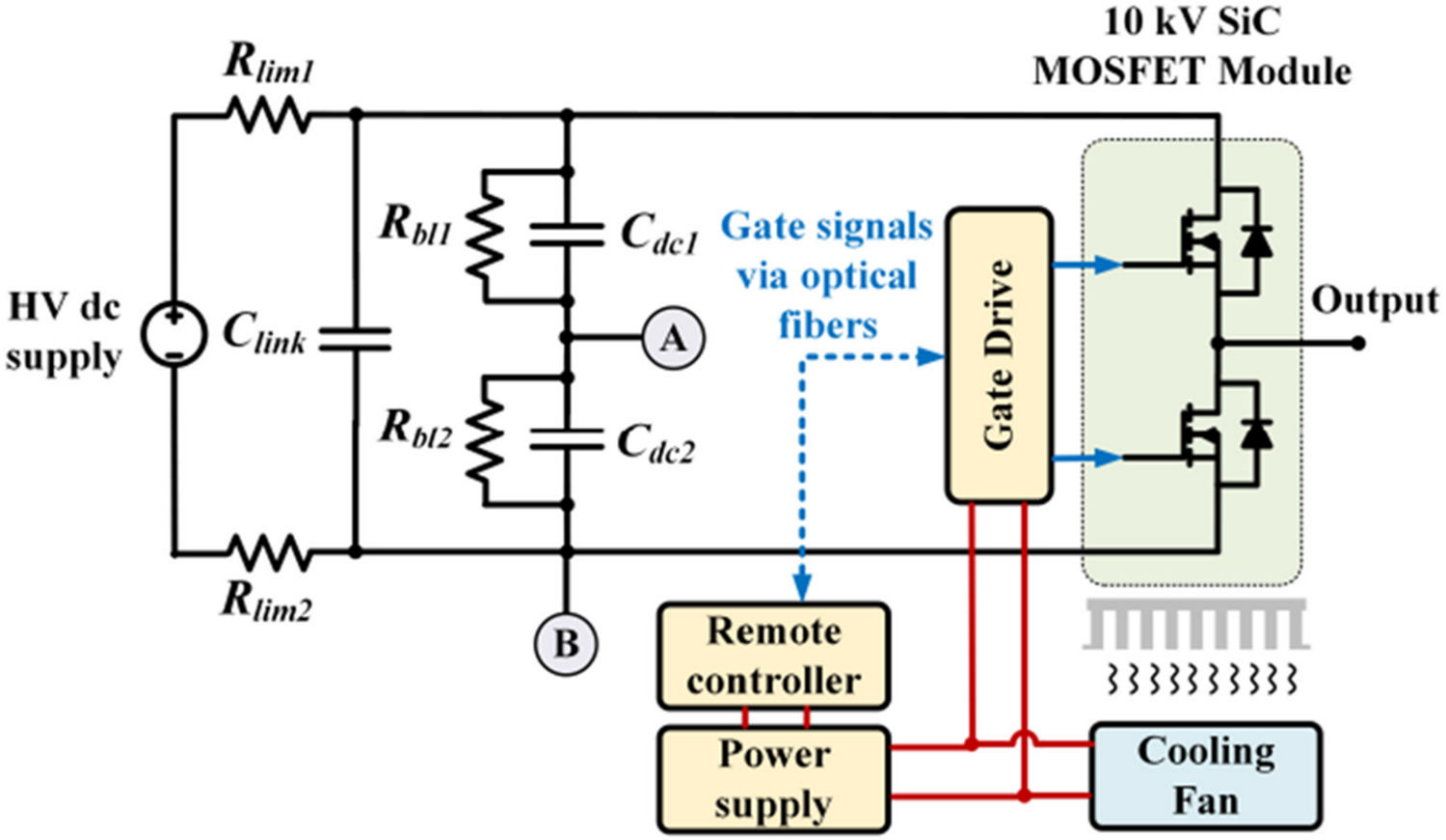
Circuit diagram of the voltage pulse generator.

**FIGURE 3.**
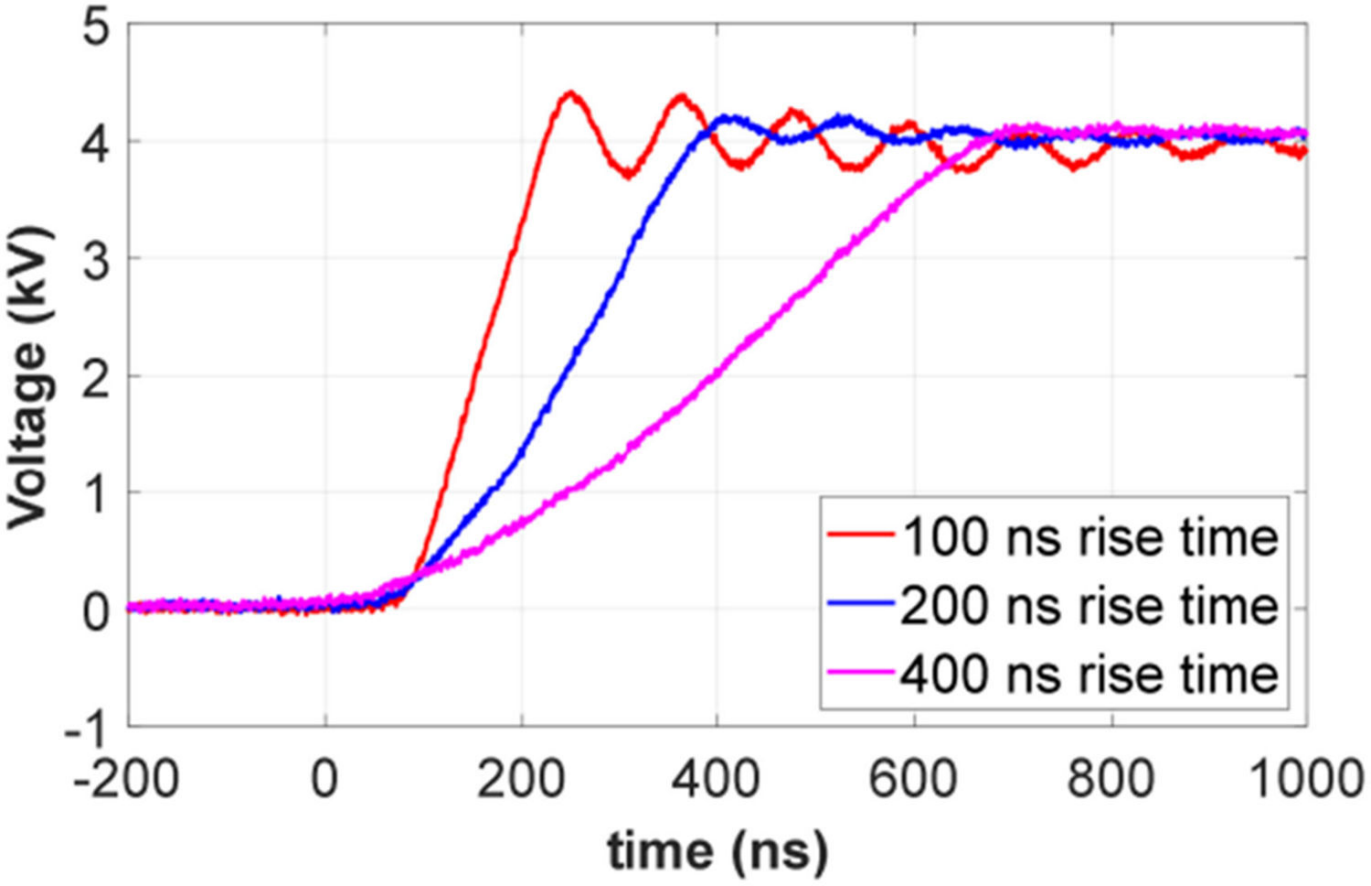
Voltage pulse waveforms applied to the winding sample.

The circuit diagram of the voltage pulse generator is shown in Fig. 2. The 10 kV SiC device used is a customized XHV-6 MOSFET module from Wolfspeed. *R_lim1_* and *R_lim2_* are current limiting resistors protecting the test platform in case of a catastrophic insulation failure of the test sample. *R_bl1_* and *R_bl2_* are voltage balancing resistors for *C_dc1_* and *C_dc2_*. The dc bus voltage on *C_link_* can be up to 7 kV with 10 kV devices. Therefore, the generator output can be up to 7 kV (unipolar) or ±3.5 kV (bipolar). The output voltage polarity can be changed by adjusting the grounding point from A to B or vice versa. The voltage rise/fall time of the generator output is associated with the capacitance of the test sample. The motor winding sample capacitance was measured as 170 pF using an impedance analyzer. With this sample capacitance, the applied voltage pulse can have a voltage rise/fall time as short as 100 ns. The voltage rise/fall time can be adjusted by changing the gate resistors in the gate drive. Fig. 3 shows generator outputs with different rise times applied to the winding sample. The dc bus voltage was 4 kV. The maximum voltage overshoot occurred at the rise time of 100 ns and the voltage increase was 381 V. The maximum switching frequency of the voltage pulse generator is 10 kHz, which is limited by its cooling system.

### B. MOTOR WINDING SAMPLE

**FIGURE 4.**
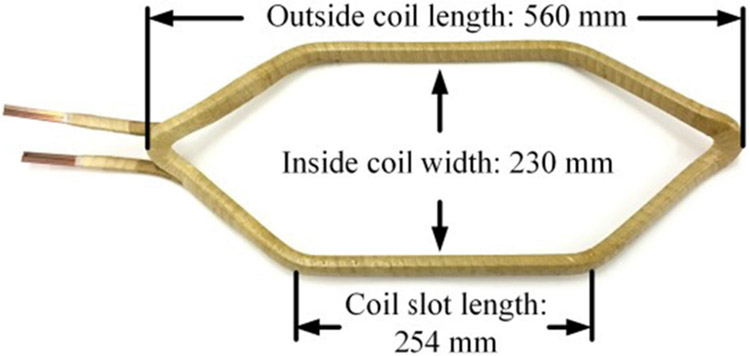
Dimensions of the 2 kV form-wound coil sample.

**FIGURE 5.**
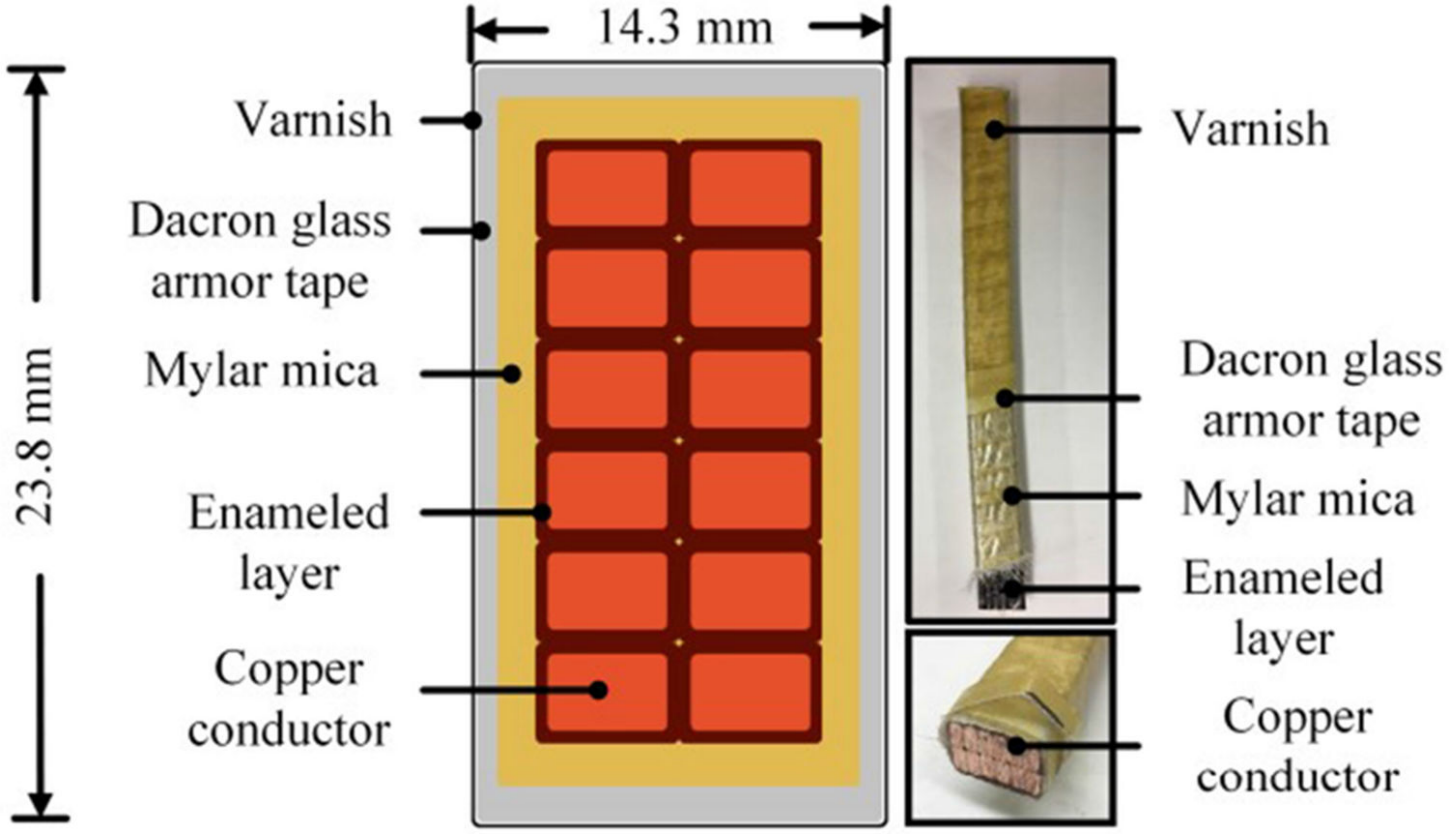
A cross-sectional view of the form-wound coil.

The motor winding sample is a 2 kV rated form-wound coil fabricated by a commercial motor manufacturer. The coil is for traditional 60 Hz induction machines (not driven by VSDs). Its dimensions are shown in Fig. 4. Fig. 5 depicts four insulation layers of the coil. The enameled layer mainly serves as the insulation between conductor strands. The ground-wall insulation consists of mylar mica tape, dacron glass armor tape, and varnish. During partial discharge tests, the form-wound coil resides in a linear motor stator as shown in Fig. 1. The stator base has no insulation coating and is always grounded.

**FIGURE 6.**
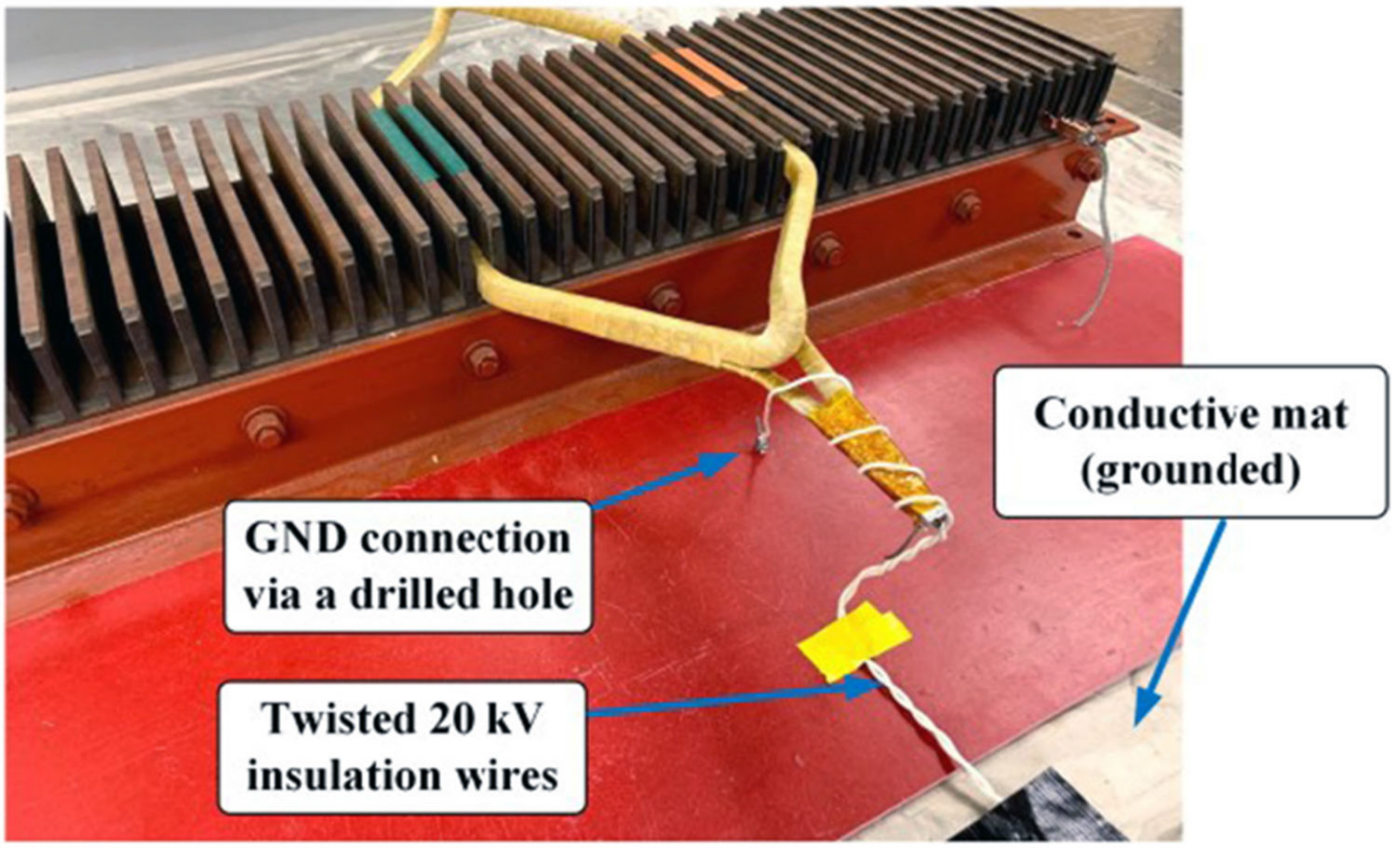
Connection to the test sample.

The connection between the voltage pulse generator and the winding sample was made of 92 cm 20 kV rated insulation wires as shown in Fig. 6. The wires are twisted to reduce the connection inductance. One insulation wire was clamped to the winding sample using a small metal clip. The connection clip was then wrapped by aluminum foils to cover its sharp edges and corners. Three layers of 3.5 mil Kapton tapes were attached to the external surface of the aluminum foil.

### C. MEASUREMENT DEVICES

Measurement devices include a 100 MHz 6 kV voltage probe (p/n: THDP0100) measuring the voltage across the test sample and two antennas (p/n: NMOQ) for partial discharge detection. Two antennas are used as shown in Fig. 1. This is to cross-check the partial discharge detection results. The antenna has a bandwidth from 136 to 960 MHz. Each antenna connects to the oscilloscope (p/n: MDO 4104) through a 500 MHz high-pass filter (p/n: CHPFL-0500). This is to further suppress background noises and switching noises from the voltage pulse generator, as suggested in [30]-[32].

**FIGURE 7.**
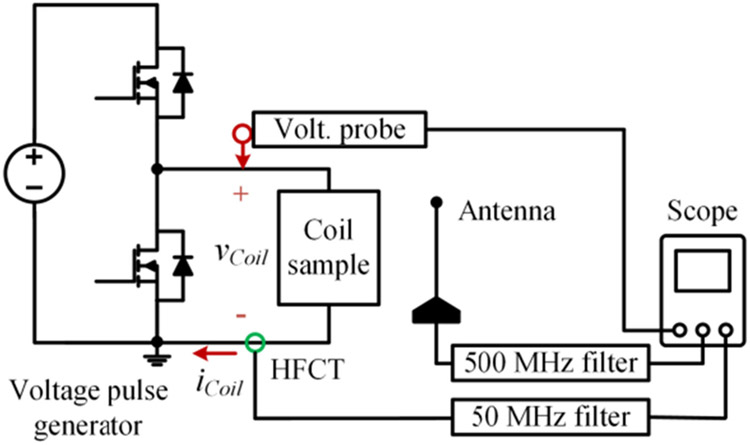
Circuit diagram for evaluating antenna-based detection method.

**FIGURE 8.**
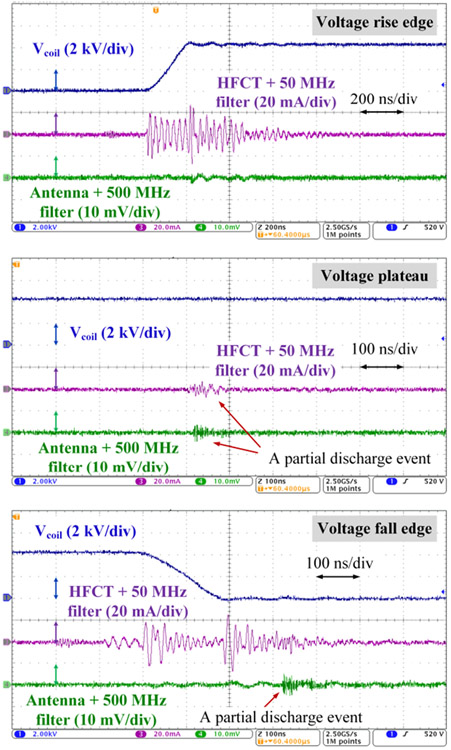
Test waveforms with a 4.3 kV voltage pulse applied to the test sample.

Detecting partial discharges during fast switching edges is challenging due to the interference of the switching noises. Effectiveness of the antenna-based detection was checked by comparing against detection results made by a high-frequency current transducer (HFCT) which is commonly used in existing partial discharge studies. The test circuit diagram is shown in Fig. 7. The HFCT (p/n: Pearson 2877) has a bandwidth up to 200 MHz and connected to the oscilloscope via a 50 MHz high-pass filter (P/N: CHPFL-0050). Fig. 8 shows test results when a 4.3 kV voltage pulse was applied to the test sample. At the voltage rise edge, the HFCT channel had severe oscillations caused by the displacement current flowing through the sample capacitance under high dv/dt. This switching noise had a magnitude of 2 mV in the antenna channel. During the voltage plateau, both channels captured a partial discharge. The antenna channel output was about 5 mV. At the voltage fall edge, a partial discharge was detected by the antenna, but the HFCT channel had no apparent indication. In following tests, only the antenna-based detection is used.

### D. PARTIAL DISCHARGE FREE VALIDATION

**FIGURE 9.**
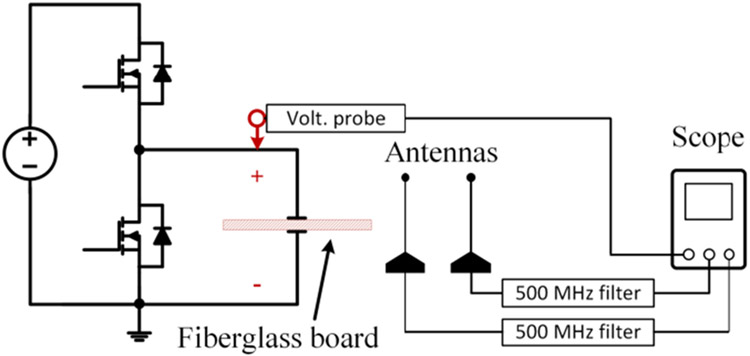
Circuit diagram for validating test setup free of partial discharges.

Validation tests were performed to check that the test setup itself is free of partial discharges. The test circuit diagram is shown in Fig. 9. The winding sample was lifted right above the stator base. A fiberglass board was inserted between them. The fiberglass board has a thickness of 6 mm. Single voltage pulses up to 5.5 kV were applied and the voltage rise/fall time was 100 ns. No partial discharge event was captured by the antennas during tests. The 5.5 kV voltage is sufficiently higher than the PDIVs of the motor winding sample, which is around 4 kV as shown in Section IV. Therefore, the validation tests were not performed at higher voltages.

## III. THREE-STEP TEST APPROACH

**FIGURE 10.**
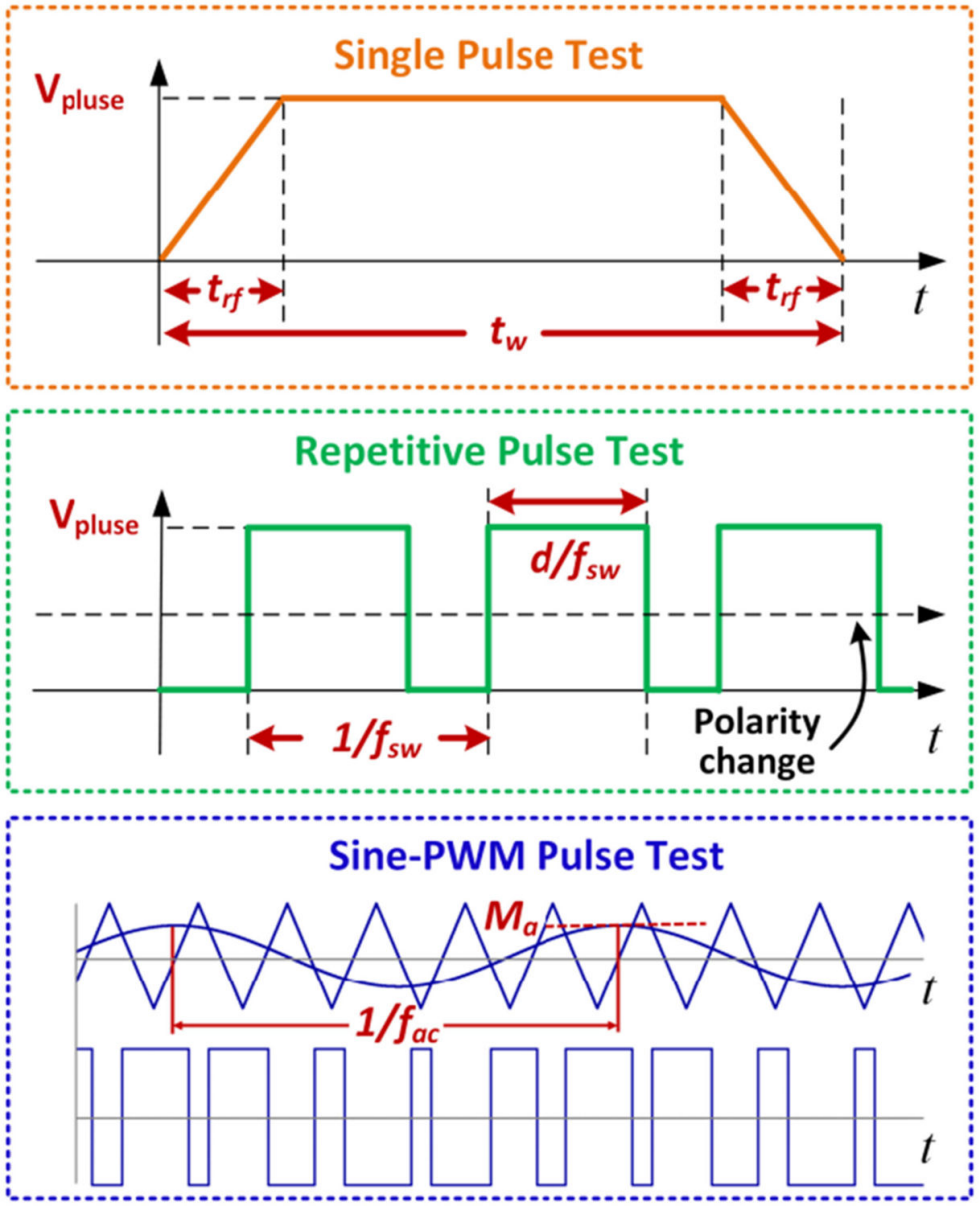
An overview of the three-step test approach.

PDIVs and RPDIVs are two key parameters indicating voltage levels at which partial discharges start to occur in an insulation system. The changes of PDIVs and RPDIVs of the winding sample under different voltage waveforms were tested using a three-step partial discharge test approach. An overview of the test approach is shown in Fig. 10 and Table I. Single pulse tests study mainly effects of voltage rise/fall and voltage pulse width. Only one voltage pulse is applied to the test sample each time. This greatly mitigates impacts such as accumulated charges, and elevated temperatures of the test sample. Repetitive tests focus on effects of pulse repetitive rate, duty ratio, and voltage polarity. Sine-PWM modulation is widely utilized in VSDs to produce sinusoidal voltages with variable amplitudes and frequencies. Voltage stress applied to the winding sample in sine-PWM pulse tests is close to real operation conditions. This sequential and systematic test approach focuses on the voltage stress experienced by a motor winding under VSDs while mitigate thermal effects, accumulated charges, and interactions of voltage waveform parameters. It is also applicable to partial discharge studies on other circuit components in VSDs (e.g., printed circuit boards, switching device package, and busbar).

### A. SINGLE PULSE TESTS

**FIGURE 11.**
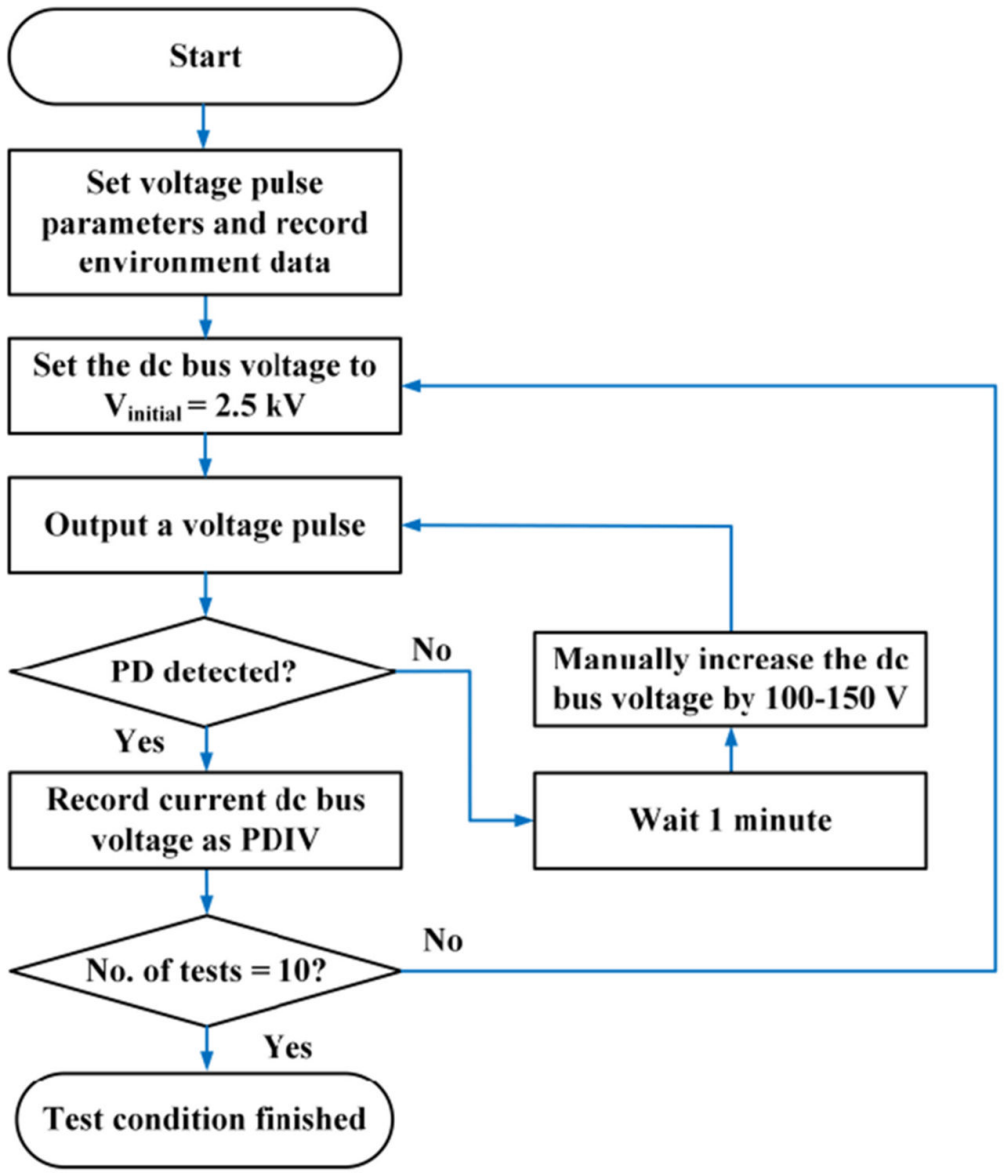
Test procedure for single pulse tests.

In single pulse test, four voltage rise/fall times were selected: 100 ns, 200 ns, 400 ns, and 800 ns. The voltage rise/fall times were selected based on switching times of medium-voltage rated SiC MOFETs and Si IGBTs. Four pulse widths were used: 10 μs, 50 μs, 100 μs, and 1000 μs. Their combination created 16 test conditions. Test procedure for each test condition is shown in Fig. 11. The environmental data include room temperature, relative humidity, and air pressure, which were recorded by an environmental meter (p/n: SD700). The time interval between two voltage pulses was at least one minute to avoid accumulation effects in the sample. Each test condition was repeated ten times to ensure the reliability of test results.

### B. REPETITIVE PULSE TESTS

Repetitive pulse tests performed in this work can be divided into three test groups. The first test group continued to investigate the effects of voltage rise/fall time since the application of SiC devices is a major focus of this work. The first test group used unipolar voltage pulses with a fixed repetitive rate (5 kHz) and a fixed duty ratio (0.5). The range of voltage rise/fall time was still from 100 ns to 800 ns. The second test group studied the effects of pulse repetitive rate and duty ratio using unipolar voltage pulses. This test group was further divided into two sets of tests. In the first set, the duty ratio of voltage pulses was fixed at 0.5 while the pulse repetitive rate was changed from 1 kHz to 8 kHz. This was to keep a fixed ratio between average and peak voltage applied to the test sample. The second test set kept the voltage pulse width constant at 100 μs. The pulse repetitive rate changed from 1 kHz to 8 kHz. The duty ratio of voltage pulses varied as the pulse repetitive rate was adjusted. The third test group studied the effects of voltage polarity. The test sample was stressed by unipolar and bipolar voltage pulses with same voltage rise/fall time (100 ns) and duty ratio (0.5). The pulse repetitive rate also changed from 1 kHz to 8 kHz in the third test group.

**FIGURE 12.**
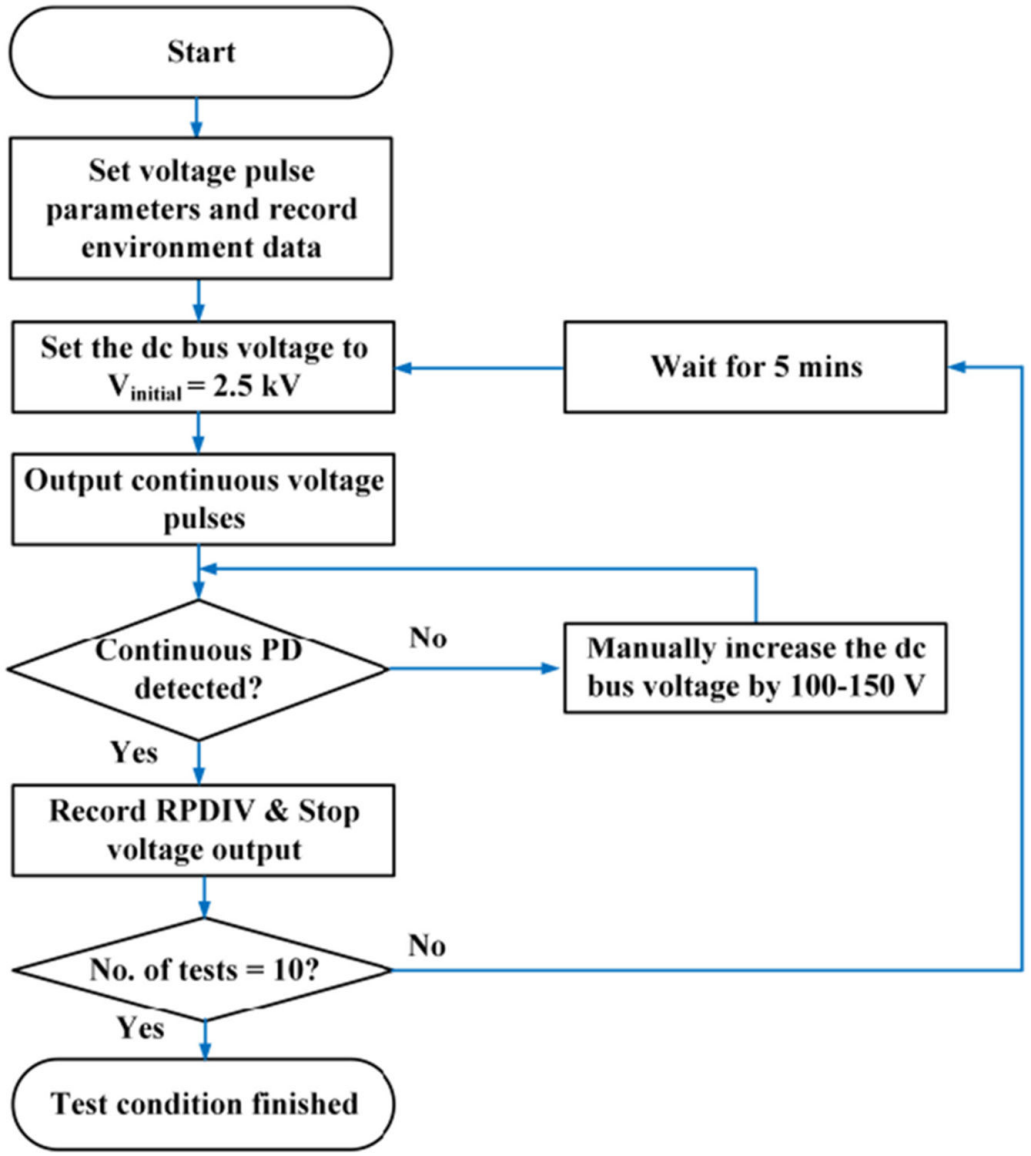
Test procedure for repetitive and sine-PWM pulse tests.

Each test condition of repetitive pulse tests followed the procedure shown in Fig. 12. In repetitive pulse tests, RPDIVs of the winding sample were measured. The criteria for RPDIVs are as follow. Partial discharges were detected by monitoring the outputs of antennas. The scope was triggered by the antenna output and the trigger level was set at 2.5 mV. At each output voltage level, the antenna output was monitored for at least one minute. If the scope was triggered continuously, the current dc bus voltage would be recorded as a RPDIV. If the scope was not triggered or only triggered intermittently, the dc bus voltage would not be regarded as a RPDIV and would increase by 100-150 V to elevate voltage stress on the test sample. Each test condition was also repeated ten times. The time interval between test conditions was at least five minutes to reduce accumulated charges and dielectric heating of the test sample.

### C. SINE-PWM PULSE TESTS

The sine-PWM pulse tests focused on the effects of fundamental frequency (*f_ac_*) and modulation index (*M_a_*) of sine-PWM modulation. The tests were performed with bipolar voltage pulses with fixed voltage rise/fall time (100 ns) and repetitive rate (5 kHz). Three fundamental frequencies were used in the tests: 10 Hz, 60 Hz, and 400 Hz. Two modulation index were used: 0.1 and 0.9. Test procedure in Fig. 12 was also followed in sine-PWM pulse tests.

## IV. PARTIAL DISCHARGE TEST RESULTS

### A. SINGLE PULSE TESTS

**FIGURE 13.**
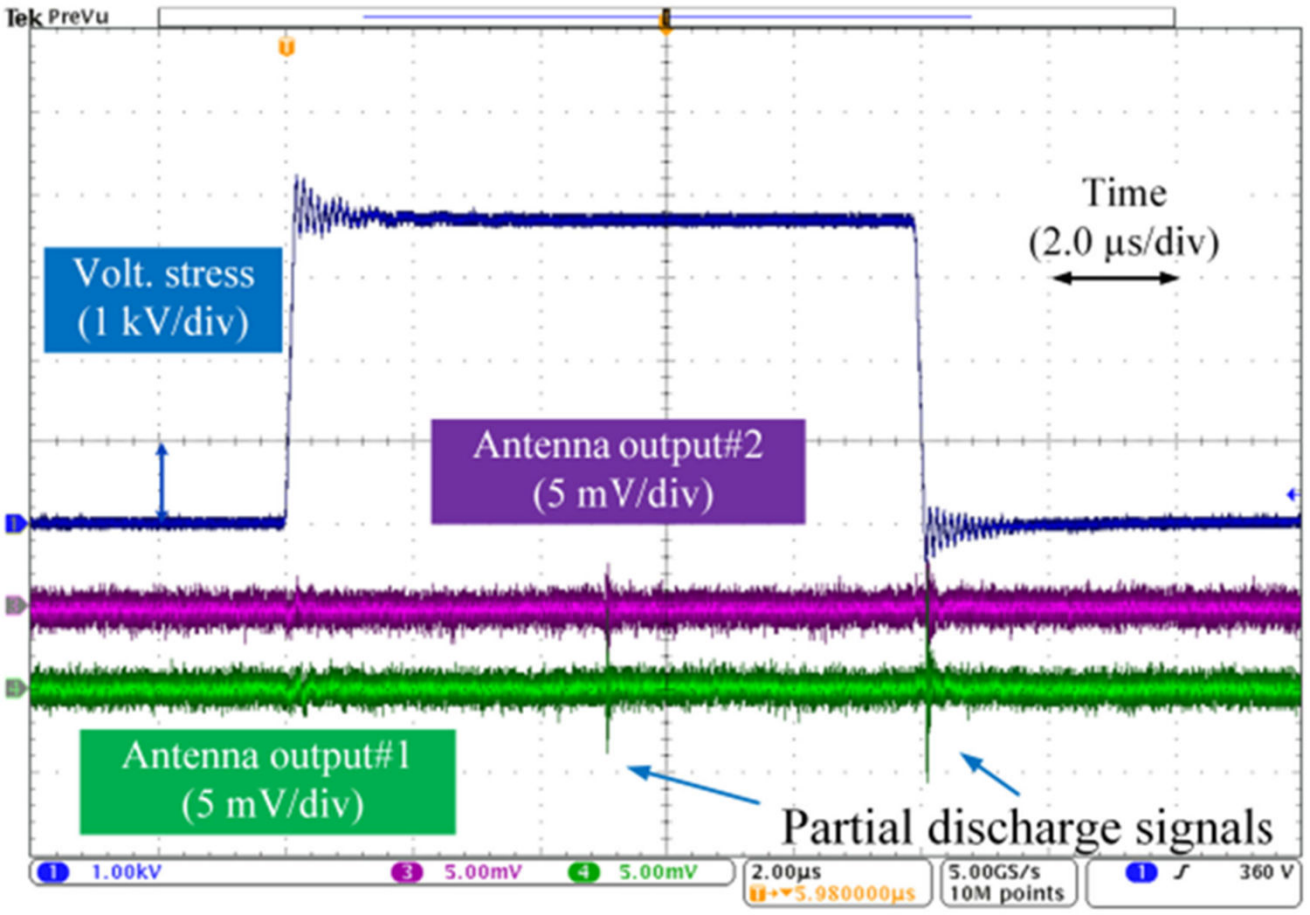
A partial discharge test waveform example under single voltage pulse.

**FIGURE 14.**
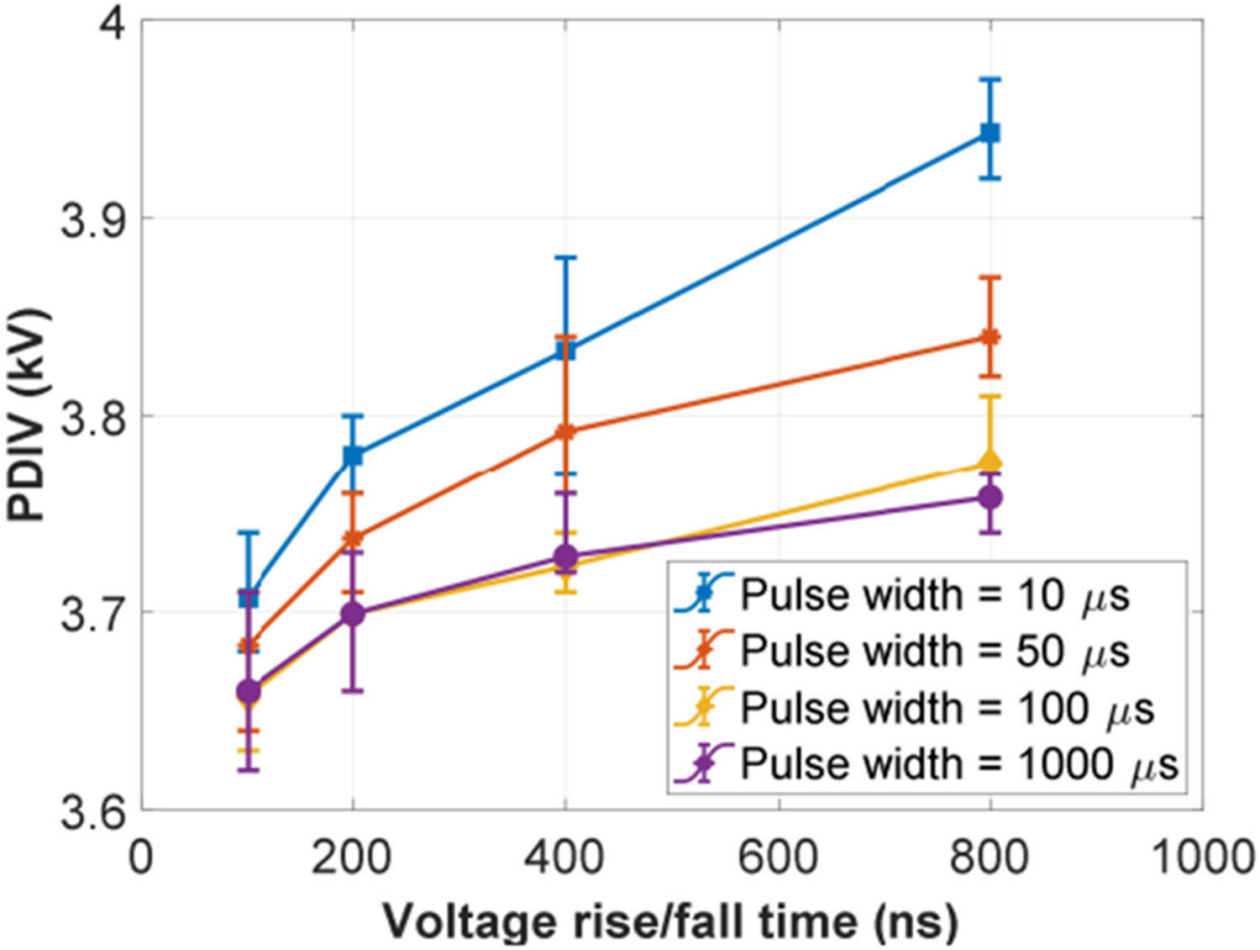
PDIVs of the test sample under single voltage pulses.

An example of single pulse test result waveforms is shown in Fig. 13, which was captured when voltage rise/fall time was 100 ns, voltage pulse width was 10 μs, and partial discharges occurred at 3.7 kV. Fig. 14 shows PDIV measurement results of single pulse tests. The solid dot is the average value of ten PDIV results of each test condition. The error bar indicates the minimum and maximum values among the ten PDIV results. Test results were recorded at environmental conditions: room temperature ranging from 15.8 °C to 17.6 °C, relative humidity ranging from 19.3% to 20.0%, and air pressure ranging from 985.3 mBar to 996.7 mBar. As shown in Fig. 14, PDIV of the test sample reduces as the voltage rise/fall time decreases. For instance, when the voltage pulse width is 10 μs, PDIV (average value) of the test sample reduces 6.5% from 3.95 kV to 3.71 kV as the voltage rise/fall time decreases from 800 ns to 100 ns. This indicates that fast switching speeds of SiC devices make partial discharges occur at lower voltages and have a negative impact on the winding insulations. Another observation is that PDIVs of the test sample tend to decrease when a long voltage pulse is applied. When the pulse width is over 100 μs, the change of PDIVs is negligible. This observation can be qualitively explained as follows. The occurrence of a partial discharge event is associated with the amount of free electric charges generated by the air ionization process. This will be illustrated in detail in Section V.A. The generation rate of free electric charges is governed by the electric field strength which is decided by the external voltage stress. When the voltage pulse width is short, a high external voltage stress is needed to make the number of free charges reach a critical value and create a partial discharge event. Moreover, a literature [33] has reported that the relationship between PDIVs and voltage pulse widths follows inverse power law approximately. It indicates that the change of PDIVs will get smaller at longer pulse width.

### B. REPETITIVE PULSE TESTS

**FIGURE 15.**
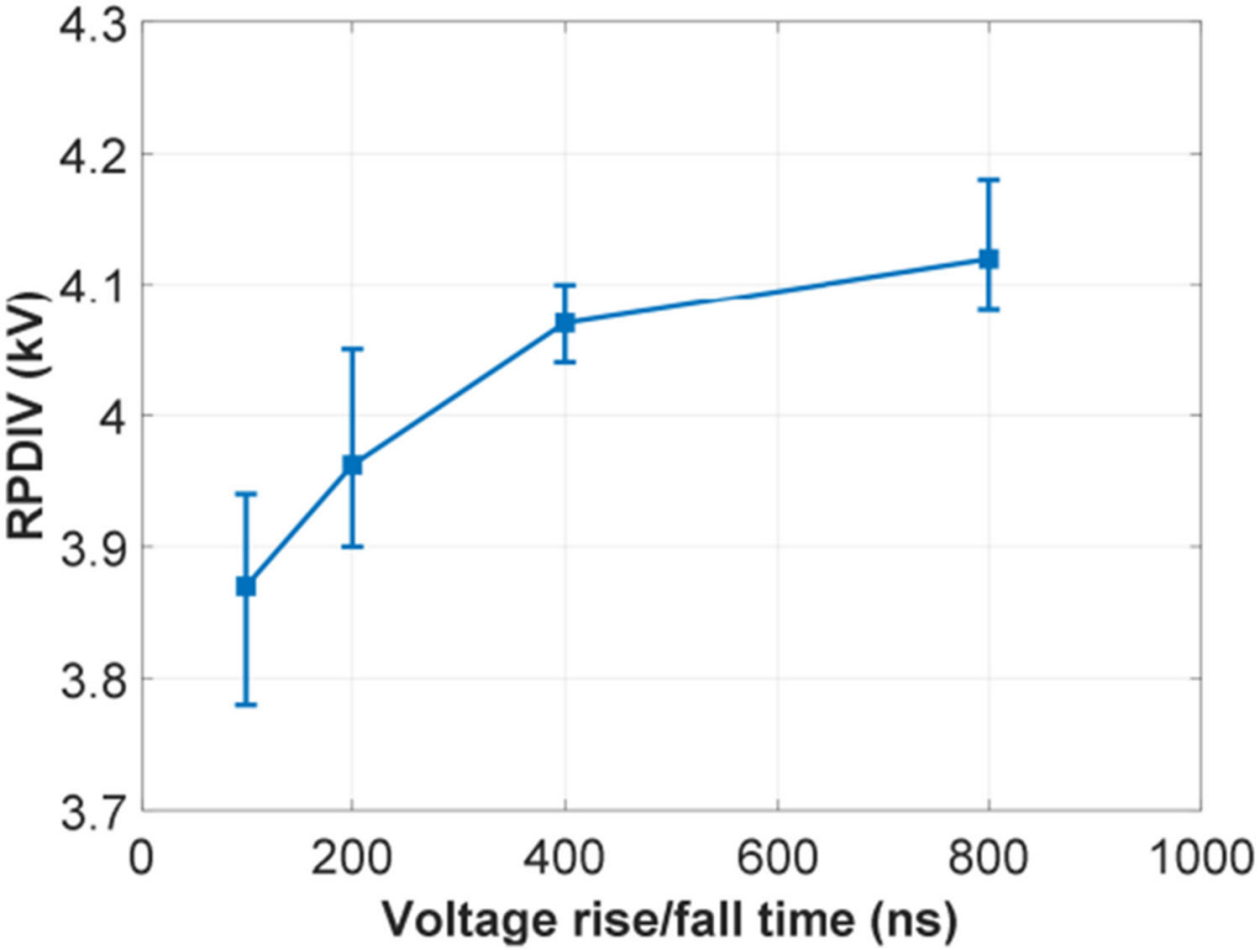
Effects of voltage rise/fall time in repetitive pulse tests (*f*_*sw*_ = 5 kHz, *d* = 0.5, and unipolar voltage).

There were three test groups in repetitive pulse tests. The first group investiaged the effects of voltage rise/fall time. The test results are shown in Fig. 15. Environmental conditions for Fig. 15 were: temperature ranging from 17.0 °C to 17.6 °C, humidity ranging from 20.3% to 21.4%, and air pressure ranging from 995.3 mBar to 996.6 mBar. Voltage rise/fall time has a similar effect in repetitive pulse tests. RPDIV of the test sample also reduces when a short voltage rise/fall time is used. Another observation is that RPDIVs are higher than PDIVs with the same voltage rise/fall time and pulse width (100 μs). This is because that RPDIVs in repetitive pulse tests not only required partial discharges to occur, but also occur continuously. The continuous partial discharges required a voltage higher than the PDIV applied to the test sample.

**FIGURE 16.**
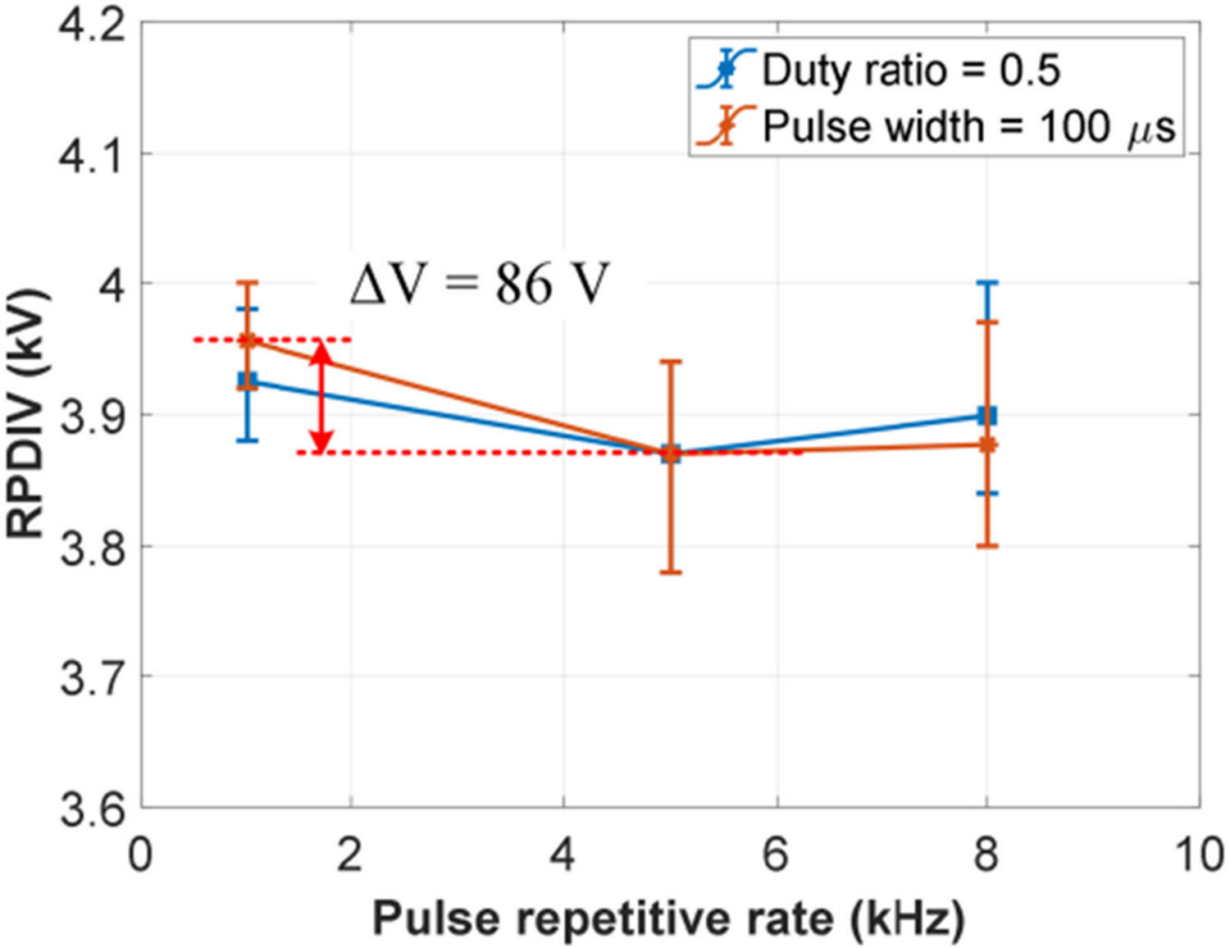
Effects of pulse repetitive rate and duty ratio in repetitive pulse tests (*t_rf_* = 100 ns and unipolar voltage).

The second test group focused on the effects of pulse repetitive rate and duty ratio. The voltage rise/fall time was fixed at 100 ns. (This voltage rise/fall time was used for all following tests.) The test results are shown in Fig. 16. The environmental data during the tests were: temperature ranging from 17.6 °C to 18.0 °C, humidity ranging from 21.2% to 21.5%, and air pressure ranging from 995.8 mBar to 997.2 mBar. Compared with voltage rise/fall time, pulse repetitive rate and duty ratio are weak affecting factors within studied parameter ranges. The variation of RPDIVs (average values) of the test sample is less than 90 V.

**FIGURE 17.**
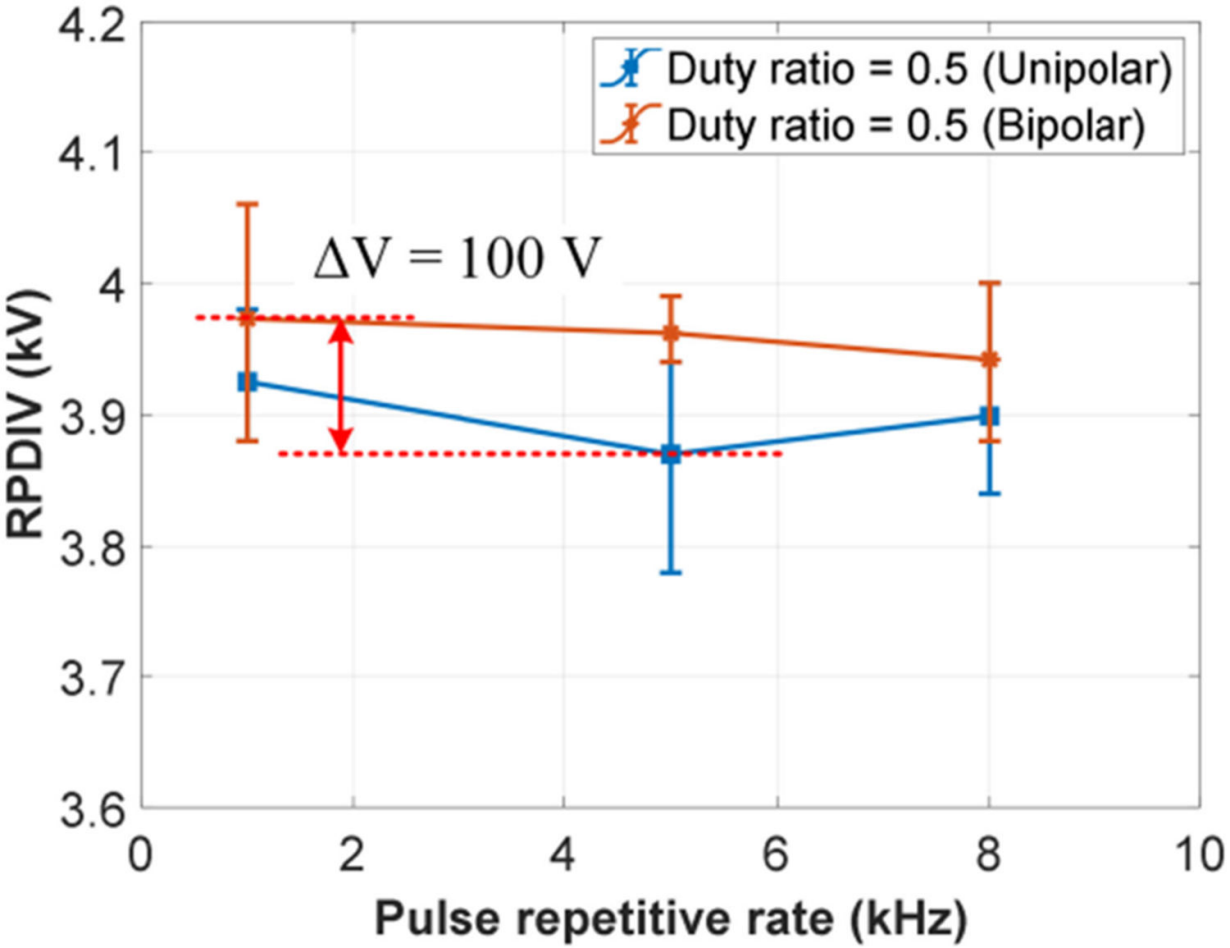
Effects of voltage polarity in repetitive pulse tests (*f_sw_* = 5 kHz and *t*_*rf*_ = 100 ns).

The effects of voltage polarity were studied in the third test group as shown in Fig. 17. The magnitude of the bipolar voltages is defined as the peak-to-peak values (ignoring voltage overshoots). RPDIVs under unipolar and bipolar voltage pulses are close, which agrees with conclusions in previous publications [34]. Bipolar test results are slightly higher than unipolar test results. The possible reason is that the unipolar voltage pulses with higher absolute values might cause more electric charge accumulations and reduce RPDIVs. The recorded environmental data were temperature ranging from 17.6 °C to 18.1 °C, humidity ranging from 21.2% to 21.6%, and air pressure ranging from 995.8 mBar to 997.5 mBar.

### C. SINE-PWM PULSE TESTS

**FIGURE 18.**
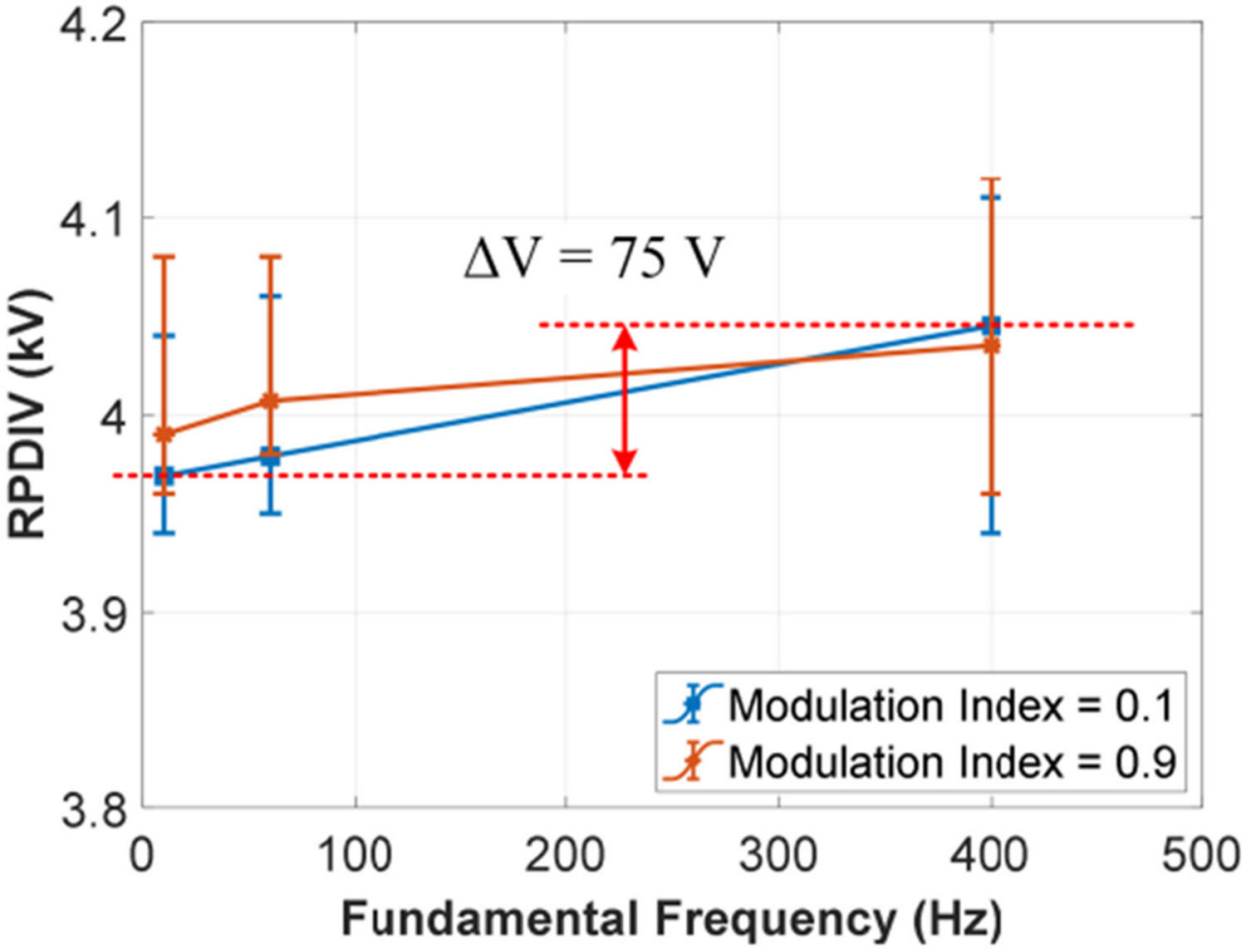
RPDIVs of the test sample under sine-PWM pulses (*f*_*sw*_ = 5 kHz, *t*_*rf*_ = 100 ns and bipolar voltage).

Test results of sine-PWM pulse tests are shown in Fig. 18 and the environment conditions were temperature from 18.0 °C to 18.3 °C, humidity from 21.1% to 21.5%, and air pressure range from 997.5 mBar to 998.1 mBar. The sine-PWM waveforms with different modulation indexes represents different magnitudes of the fundamental ac voltages. The variation of RPDIVs (average value) in Fig. 18 is small, less than 75 V. This means that the magnitude of the fundamental ac voltage is a weak affecting factor. The small variation of RPDIVs can be explained by the fact that sine-PWM waveforms are repetitive voltage pulses with varied duty ratios and duty ratio is a weak affecting factor as shown in Fig. 16.

## V. ANALYSIS OF TEST RESULTS

### A. EFFECTS OF FAST VOLTAGE RISE/FALL EDGES

**FIGURE 19.**
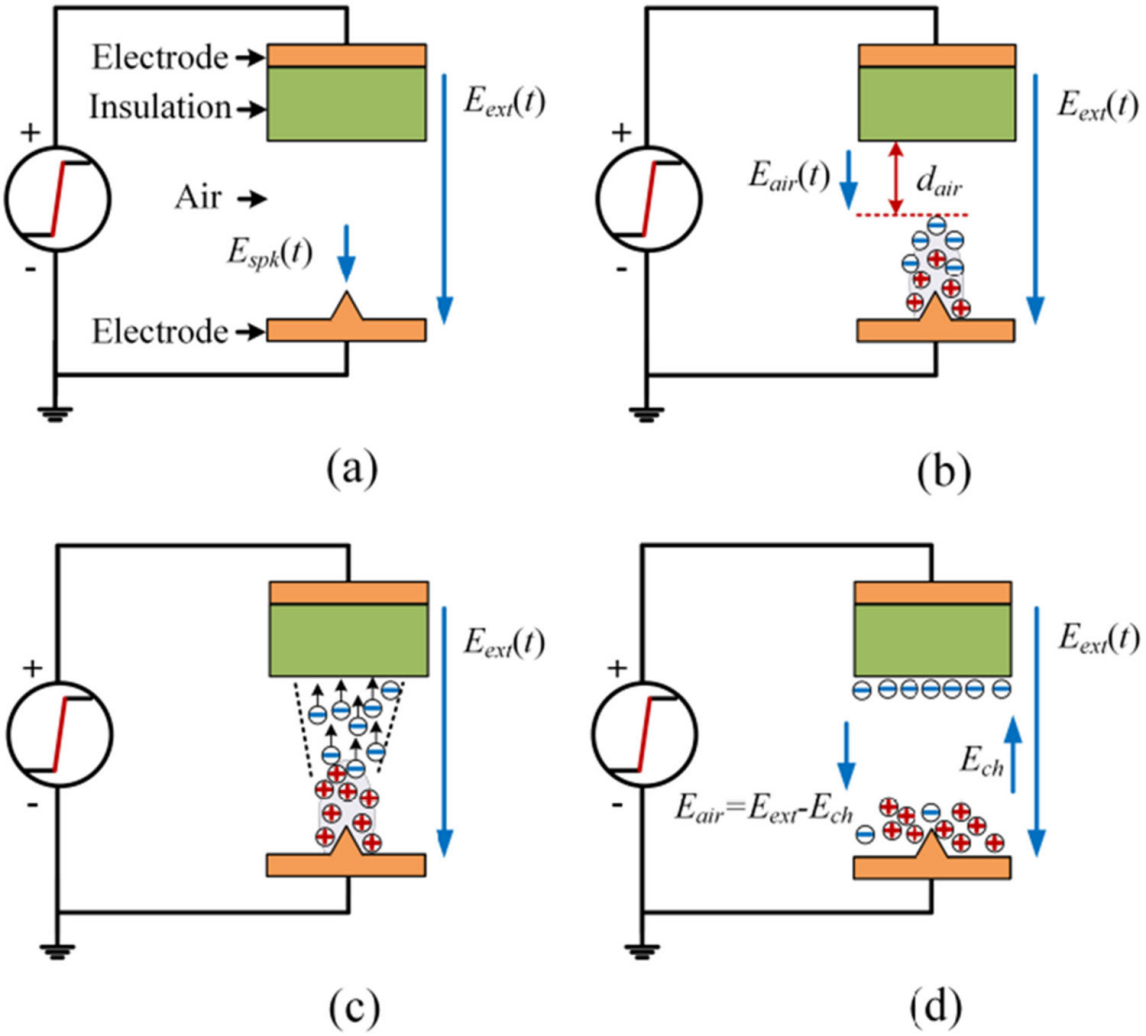
Partial discharge process during a voltage rise edge.

A hypothetical partial discharge mechanism is presented here to explain the effects of fast voltage rise/fall edges. It is inspired by analysis in [34], [35]. Fig. 19 shows the hypothetical partial discharge process during a voltage rise edge of an unipolar voltage pulse. Based on previous partial discharge studies [16], [18], [36], [37], partial discharges in a form-wound motor winding mostly likely happen in the air gap between the winding coil’s surface and the stator just outside the stator slot. Therefore, the winding sample is simplified as an electrode with an insulation layer at the top. The stator base is shown as an electrode at the bottom. The spike at the bottom electrode represents a rough edge of stator base. The air represents the small air gaps between the winding and the stator base.

- When the external voltage is applied to the test sample, an electric field (*E_ext_*) is generated in the air gap. The electric field near the spike (*E_spk_*) is higher than *E_ext_* due to the field concentration at its sharp shape. The arrows for electric fields denote their directions and spatial ranges but not magnitudes.
- As the external voltage increases and *E_spk_* exceeds a threshold value, air ionization starts at the spike area creating electrons and positive ions. Due to their smaller mass, electrons have higher mobilities and move up under *E_ext_*. This creates a shuttle-shape charge cloud. The charge cloud acts as an extension of the spike reducing the effective air gap distance (*d_air_*) and increasing the electric field in the air (*E_air_*).
- When the generated free electric charges reach a critcal amount and *E_air_* exceeds the breakdown electric field strength of air, electrons will continue propogating towards the insulation layer. A low-impedance channel is formed in the air and a large number of electrons transport towards the insluation layer. A partial discharge event occurs.
- When electrons reach the insulation layer, they attach to its surface. These electrons and positive ions which float near the spike area create an electric field (*E_ch_*) with a direction opposite from *E_ext_*. This reduces *E_air_*. Moreover, the partial discharge event depletes avaialable electrons in the spike area. These two factors stops the partial discharge process.

**FIGURE 20.**
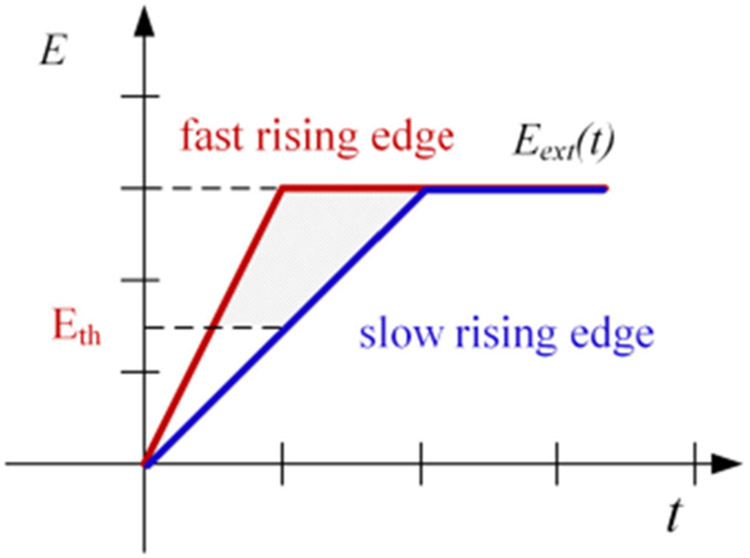
Comparison between fast and slow voltage rise edges.

Fig. 20 shows a comparison between fast and slow voltage rising edges. In the figure, E_th_ denotes the threshold electric field strength that effective air ionization starts. The occurace of a partial discharge event is associated with the the amount of free electric charges generated by the air ionization process. With a same voltage magnitude, a fast voltage rising edge provides more time and higher electric field strength for the air ionization process. This can generate more free electric charges and make partial discharges easy to occur.

**FIGURE 21.**
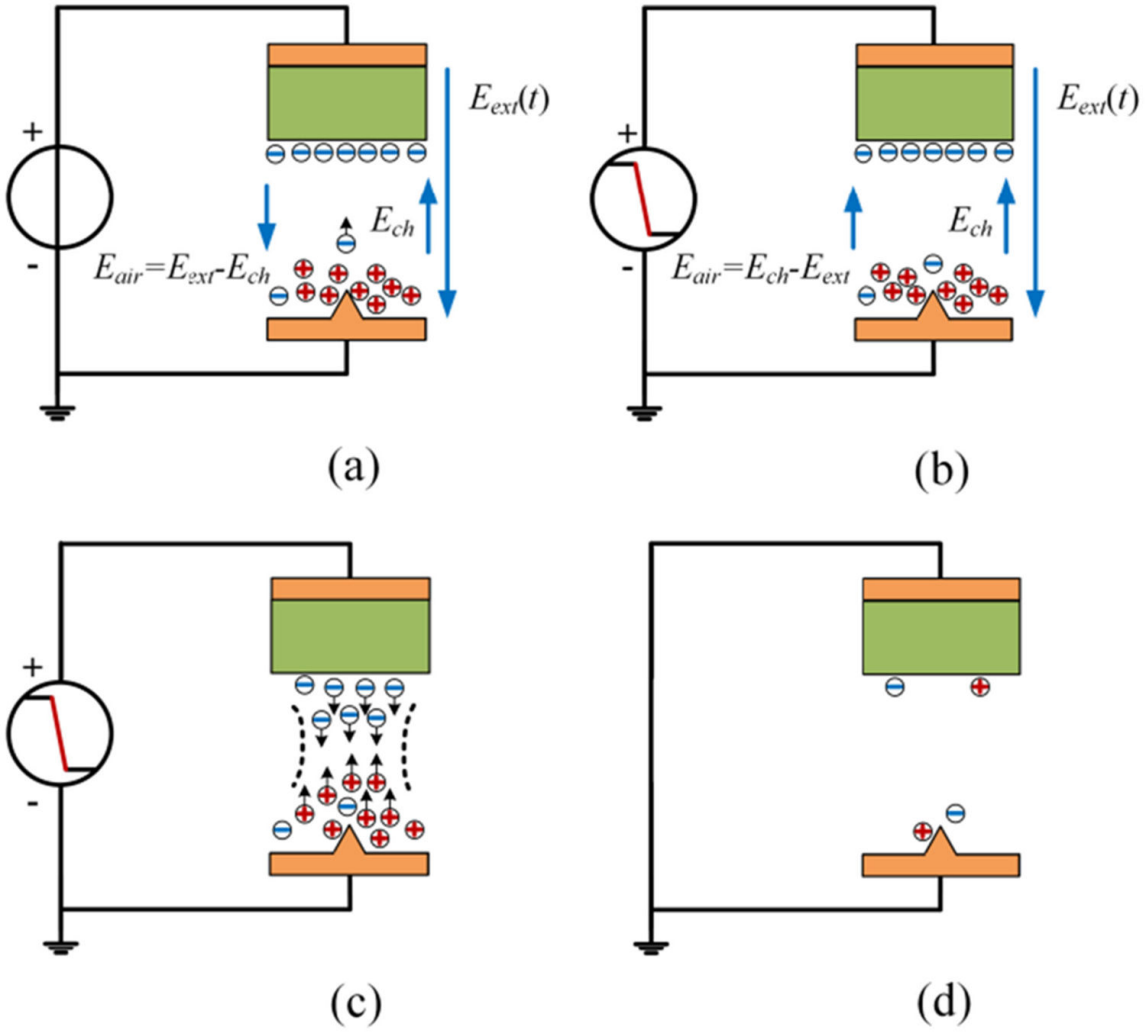
Partial discharge process during a voltage fall edge.

Fig. 21 shows the partial discharge process during the voltage fall edge.

- Before the voltage fall edge, electrons and positive ions exist in the air gap. They are from the air ionization and electron movement in step (b) of Fig. 19 (active electrons can reach the insulation layer eventually) or the partial discharge process in step (c) of Fig. 19. Electrons and positive ions are being generated continuously by the air ionization. They also decline by the recombination process or the absorption into the insulation layer. The generation and decline of electrons and ions reach an equilibrium.
- When the external voltage starts to decrease, the electric field produced by electric charges (*E_ch_*) becomes the dominant part of *E_air_* since the charge decline is a slower process compared to the voltage fall edge.
- If *E_air_* can be over the breakdown electric field strength of air (*E_bk_*), a partial discharge event occurs. Electrons and ions travel through the created low impedance channel and recombinate when they meet.
- After the partial discharge process, little charges remain in the air gap and *E_air_* is almost zero.

**FIGURE 22.**
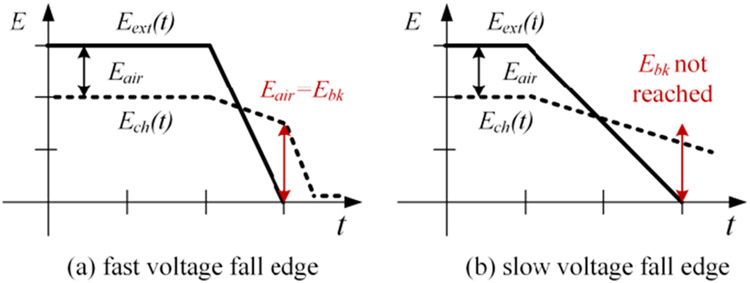
A comparison between fast and slow voltage fall edges.

Fig. 22 shows a comparison between electric fields during fast and slow voltage fall edges. The electrons and ions decline more during a slow voltage fall edge. As a result, *E_air_* is not high enough to break down the air gap and a partial discharge event does not occur.

### B. EMPIRICAL EQUATION FOR PDIVS

**FIGURE 23.**
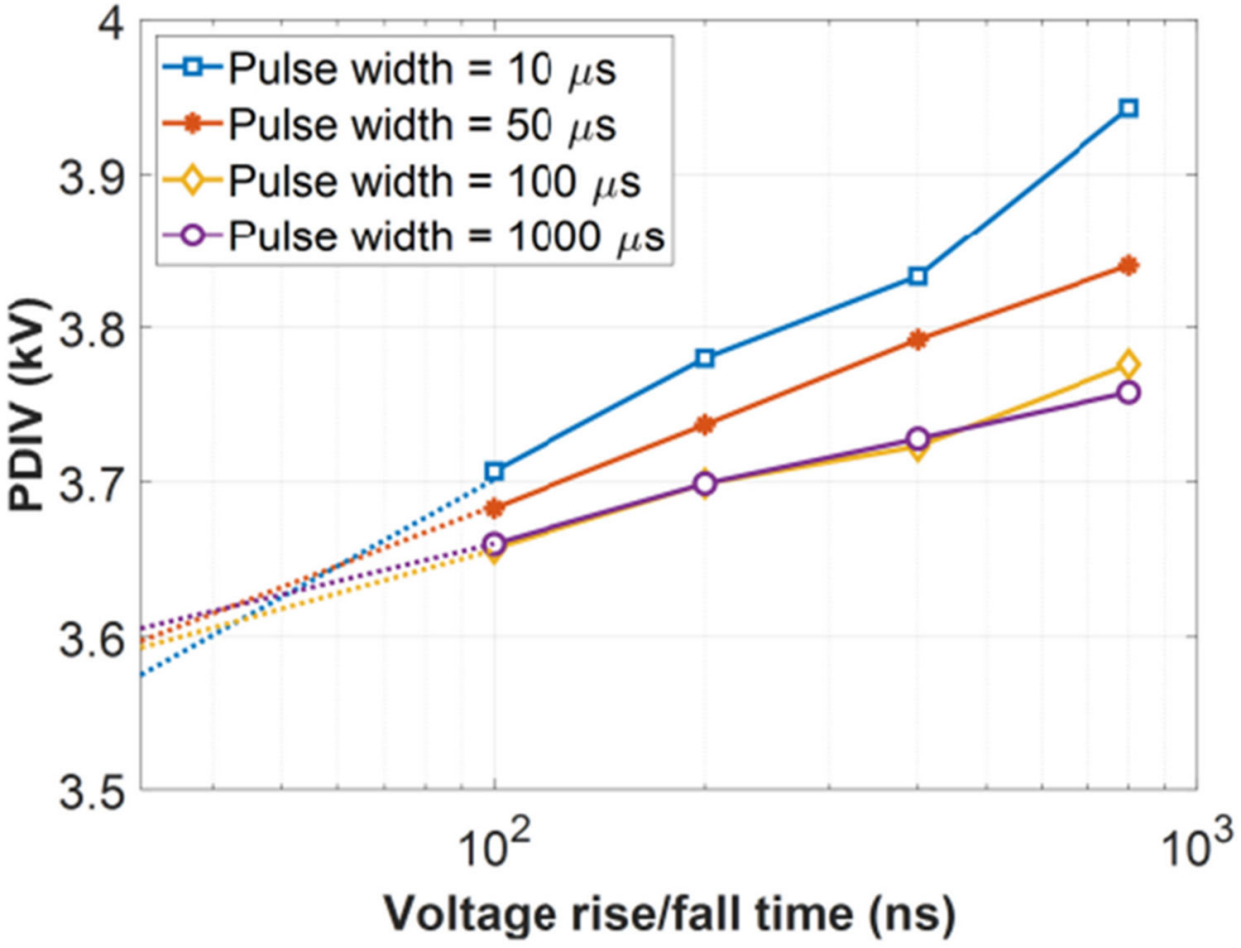
Single pulse test results with linear interpolations.

An empirical equation is proposed to estimate PDIVs based on single pulse test results. Fig. 23 shows single pulse results (average value) with x-axis (voltage rise/fall time) in log scale. The solid lines are test results and the dashed lines are their linear-interpolated extensions. The test results appear as linear curves with a log-scaled x-axis. Moreover, four curves meet approximately at a joint point (50 ns, 3.6 kV). A possible reason of this joint point is that when the voltage rise/fall time is shorter than 50 ns, it becomes a dominant factor excluding the effects of the voltage pulse width.

Based on these two observations, equation (1) is proposed to estimate PDIVs under various voltage rise/fall time and pulse width conditions. *t_rf_* is the voltage rise/fall time (in ns) and (x_0_, y_0_) is the junction point. The slope of the curve *k* is a function of the voltage pulse width *t_w_* (in μs). In Fig. 23, the curve slope reduces with increased voltage pulse width and becomes stable after the pulse width is over 100 μs. An exponential function is used to express this trend as shown in (2).

(1) $$PDIV\;(t_{rf},t_{w})=k\;(t_{w})\cdot [\text{ln}\;(t_{\text{rf}})-x_{0}]+y_{0}$$

(2) $$k\;(t_{w})=a\cdot e^{-\frac{t_{w}}{t_{0}}}+b$$

**FIGURE 24.**
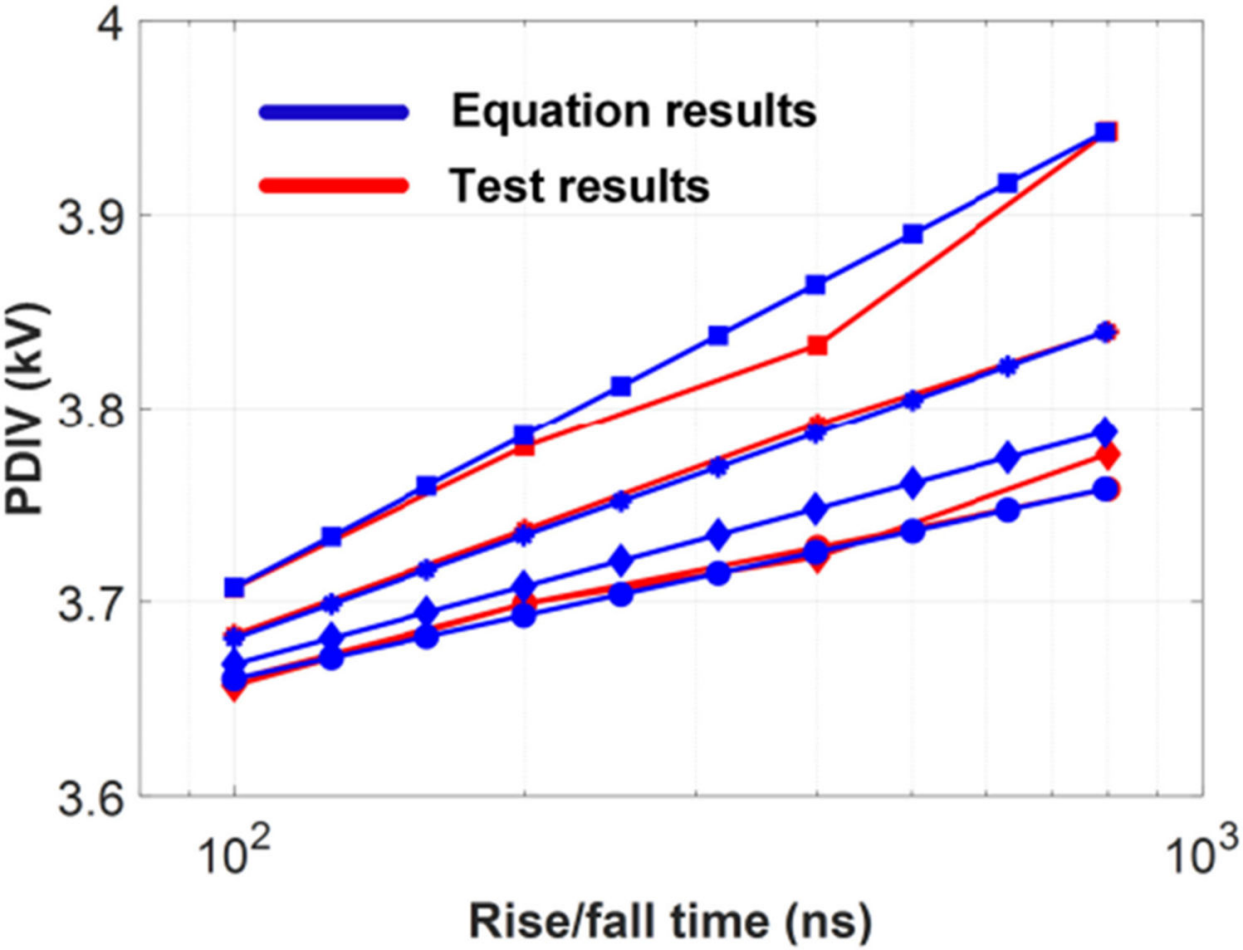
Comparison between test results and equation calculation results.

Equation coefficients were calculated using a curve fitting software. The results are listed in Table II. The coefficient of determination R^2^ of the curve fitting result is 0.976. A comparison between test and equation results is shown in Fig. 24. This empirical equation shows that PDIVs of a form-wound winding have an approximate logarithmic relationship with voltage rise/fall time.

## VI. CONCLUSION

This paper studies partial discharges for the phase-to-ground insulation of a medium-voltage form-wound winding. A test platform featuring 10 kV SiC devices is built which can stress test samples with high dv/dt voltage pulses. A three-step test approach is presented to investigate systematically effects of voltage rise/fall time, voltage pulse width, voltage polarity, pulse repetitive rate, duty ratio, and fundamental frequency and modulation index of sine-PWM voltage waveforms. The voltage rise/fall time is identified as a major factor affecting the PDIVs and RPDIVs of the test sample (up to 6.5%). While other parameters have less impacts (less than 3%). This suggests that fast switching speeds of SiC devices can adversely affect the motor winding insulation. The effects of voltage rise/fall edges on partial discharge processes are qualitatively explained. An empirical equation is proposed to estimate PDIVs at various voltage rise/fall time and pulse width conditions. Test approach, test results, and analysis in this paper can be useful for manufacturers and researchers of electric machines, motor drives, and other apparatus associated with applications of SiC devices.

## Figures and Tables

**TABLE 1.** Three-step Test Approach for Partial Discharge Study

Test step	Studied Parameters
Single pulse tests	Voltage rise/fall time (*t_rf_*) Voltage pulse width (*t_w_*)
Repetitive pulse tests	Voltage pulse repetitive rate (*f_sw_*) Duty ratio (*d*) Voltage polarity (unipolar or bipolar)
Sine-PWM pulse tests	Fundamental frequency (*f_ac_*) Modulation index (*M_a_*)

**TABLE 2.** Curve Fitting Results of Equation Coefficients

Coefficients	Values
*a*	0.081
*b*	0.047
*t_0_*	49.16
*x_0_*	3.90
*y_0_*	3.63
